# Supplementary material for: Accuracy of computer-aided chest X-ray in community-based tuberculosis screening: Lessons from the 2016 Kenya National Tuberculosis Prevalence Survey
Source: PLOS Glob Public Health. 2022 Nov 23;2(11):e0001272. doi: 10.1371/journal.pgph.0001272 (PMC10022380; doi:10.1371/journal.pgph.0001272)
Supplement: S1 Text — (PDF) [file pgph.0001272.s001.pdf]

	EDUCATION QUALIFICATION	WORK EXPERIENCE
**1**	DIPLOMA IN CLINICAL MEDICINE AND SURGERY	4 YEARS
**2**	DIPLOMA IN CLINICAL MEDICINE AND SURGERY	2 YEARS
**3**	DIPLOMA IN CLINICAL MEDICINE AND SURGERY AND COMMUNITY HEALTH	1 YEAR
**4**	DIPLOMA IN CLINICAL MEDICINE SURGERY AND COMMUNITY HEALTH	1 YEAR
**5**	DIPLOMA IN CLINICALMEDICINE SURGERY AND COMMUNITY HEALTH	2 YEARS
**6**	DIPLOMA IN CLINICAL MEDICINE AND SURGERY	1 YEAR
**7**	DIPLOMA IN CLINICAL MEDICINE AND SURGERY	1 YEAR
**8**	BSc ENVIRONMENTAL HEALTH, AND DIPLOMA IN CLINICAL MEDICINE AND SURGERY	2 YEARS
**9**	DIPLOMA IN CLINICAL MEDICINE AND SURGERY	2 YEARS
**10**	DIPLOMA IN CLINICAL MEDICINE AND SURGERY	2 YEARS
