# Supplementary material for: Accuracy of computer-aided chest X-ray in community-based tuberculosis screening: Lessons from the 2016 Kenya National Tuberculosis Prevalence Survey
Source: PLOS Glob Public Health. 2022 Nov 23;2(11):e0001272. doi: 10.1371/journal.pgph.0001272 (PMC10022380; doi:10.1371/journal.pgph.0001272)
Supplement: Attachment — Submitted filename: Response to Reviewers 6thMay.docx [file pgph.0001272.s003.docx]

**PGPH-D-22-00030
Accuracy of computer-aided chest X-ray screening in the Kenya National Tuberculosis Prevalence Survey
PLOS Global Public Health**

Thank you to members of the PLOS Global Public Health editorial board for highlighting that our manuscript may be of interest to the readers and for the opportunity to address points raised by reviewers. Please find below a point by point response to reviewers. Please also find attached the manuscript with track changes and the clean version. The Line numbers on the responses is from the un-marked version “Manuscript”. We look forward to your feedback.

**Journal Requirements:**

1. We see that your study includes live participants, but you have not included an Ethics Statement. Please update your manuscript file to include an Ethics Statement subsection to your Materials and Methods section. It should include:

i) The full name(s) of the Institutional Review Board(s) or Ethics Committee(s)

Thank you for highlighting this. We covered this in the ethics considerations section line 237-244. We will also fill it in the ethics statement subsection.

*“This study was conducted as part of the Kenya Prevalence survey ethics approval reference number SSC 2094 by the Kenya Medical Research Institute. Prevalence survey participants provided written informed consent for survey activities. Additional approval was obtained from the Division of National Tuberculosis, Leprosy and Lung Disease program which is the custodian of the TB prevalence survey data.”*

2. In the online submission form, you indicated that "The Kenya National Tuberculosis, Leprosy and Lung Disease program is the custodian of the 2016 Kenya Tuberculosis Prevalence Survey data.". All PLOS journals now require all data underlying the findings described in their manuscript to be freely available to other researchers, either 1. In a public repository, 2. Within the manuscript itself, or 3. Uploaded as supplementary information.
This policy applies to all data except where public deposition would breach compliance with the protocol approved by your research ethics board. If your data cannot be made publicly available for ethical or legal reasons (e.g., public availability would compromise patient privacy), please explain your reasons by return email and your exemption request will be escalated to the editor for approval. Your exemption request will be handled independently and will not hold up the peer review process, but will need to be resolved should your manuscript be accepted for publication. One of the Editorial team will then be in touch if there are any issues.

The prevalence survey data had been shared to PLOS One by the National TB program during the publication of the prevalence survey paper in the links below [**S1 Excel.**](https://journals.plos.org/plosone/article/file?type=supplementary&id=10.1371/journal.pone.0209098.s001)

**Information line data for the Kenya TB prevalence survey 2016.** <https://doi.org/10.1371/journal.pone.0209098.s001> (CSV)

**[S2 Excel.](https://journals.plos.org/plosone/article/file?type=supplementary&id=10.1371/journal.pone.0209098.s002) Data dictionary for the information line data for the Kenya TB prevalence survey 2016.**

<https://doi.org/10.1371/journal.pone.0209098.s002> (XLS)

3. Please amend your detailed Financial Disclosure statement. This is published with the article, therefore should be completed in full sentences and contain the exact wording you wish to be published.
 
ii). State the initials, alongside each funding source, of each author to receive each grant.
iii). State what role the funders took in the study. If the funders had no role in your study, please state: “The funders had no role in study design, data collection and analysis, decision to publish, or preparation of the manuscript.”

Thank you for this. We have updated the financial disclosure section as advised. See line 520-529

*This research was funded by the National Institute for Health Research (NIHR) (IMPALA, grant reference 16/136/35) using UK aid from the UK Government to support global health research. SBS was Director of the IMPALA Global Health Research Unit at the time of this work and BNM was a PhD candidate funded through IMPALA. PM was funded by Wellcome (200901/Z/16/Z). MYRH was in part supported by a strategic award from Wellcome to Malawi-Liverpool-Wellcome Trust Clinical Research programme (206545/Z/17/Z). The views expressed in this publication are those of the author(s) and not necessarily those of the National Institute for Health Research or the UK Department of Health and Social Care. The funders had no role in study design, data collection and analysis, decision to publish, or preparation of the manuscript.*

**Additional Editor Comments (if provided):**

Review your statistical model to estimate TB prevalence in participants with a very low underlying TB prevalence rate (those with no symptoms and a normal CXR). This has not been done with other publications of TB prevalence survey data and the authors have not provided any context/justification for their decision to follow this approach. Engage someone with strong knowledge of modelling to guide through section.

Thank you. The primary objective of the study was not to estimate prevalence of TB, but rather to evaluate the accuracy of the CAD4TBv6 software among the TB prevalence survey population. We used a latent class model to estimate accuracy, recognising that completion of the reference standard (microbiological testing for pulmonary TB) was conditional on participants having an abnormal chest X-ray suggestive of TB or TB symptoms. Estimates of TB prevalence by subgroup were generated as secondary output of the model, and will be necessarily slightly different from empirical prevalence survey estimates, as the primary prevalence survey did not undertake microbiological testing of all participants. We think this is a strength of this analysis, and have added additional text to the manuscript to provide further discussion of this point (LINE 442-463). Author MYRH is a biostatistician.

*“In addition, we used a latent class model to estimate accuracy, recognising that completion of the reference standard (microbiological testing for pulmonary TB) was conditional on participants having an abnormal chest X-ray suggestive of TB or TB symptoms. Though TB prevalence surveys are designed to be as accurate as possible, they are limited by cost. Extremely large prevalence surveys needed to provide reasonable precision (usually +/-10%, as per the WHO standards for prevalence survey)(20) are rarely affordable. Sputum microbiological testing is the major driver of cost in prevalence surveys, and so programmes must significantly limit the numbers of sputum tests done to make estimation of prevalence feasible. This is done by having a two-stage screening approach, recognising that some prevalent TB may be missed. In our analysis we were able to utilise all data available for all the ~62,000 participants in the prevalence survey, rather than just the ~8000 who underwent a sputum microbiological test.*

*In a programmatic prevalence survey or community-based active case finding intervention, CAD would be used as an initial screening test among all participants. As prevalence survey participants who underwent sputum testing have substantially different characteristics (Table 1) and prevalence of disease from participants who did not undergo sputum testing, non-inclusion of these participants is likely to bias estimates of accuracy. Adopting the latent class modelling approach therefore provides improved estimates of accuracy of CAD for the target population (community members participating in TB screening programmes), rather than focusing on only those whose sputum was tested. Our model prevalence estimates are slightly higher than empirical estimates obtained from the prevalence survey (especially for men: 927 per 100,000 [783-1090] in our analysis vs. 809 per 100,000 [656-962] in the weighted prevalence survey estimate) ;(17) this is to be anticipated as the primary prevalence survey did not undertake microbiological testing of all participants.”*

**Review Comments to the Author**

Please use the space provided to explain your answers to the questions above. You may also include additional comments for the author, including concerns about dual publication, research ethics, or publication ethics. (Please upload your review as an attachment if it exceeds 20,000 characters)

**Reviewer #1:** The authors present an evaluation of CAD4TB v6 software using a very large and well-characterized data set. CAD software is ideally positioned for use in large-scale, roving surveys, so this is an ideal data set with which to conduct a CAD evaluation.

However, the added-value of the Bayesian modelling to estimate the TB burden in survey participants without TB symptoms or CXR signs suggestive of TB is unclear. The prevalence survey was designed to be the most accurate measure of TB prevalence possible and this type of modelling was not done to estimate disease burden in the prevalence survey publication you cited (#19). The background section does not introduce this approach or any studies using it to estimate disease burden. In this particular context, it seems like at best, this modelling approach would provide marginal value-add because people with no symptoms and a normal CXR have an extremely low chance of having TB, and that it may introduce falsely label participants as “cases” for the analysis. Given that this data set is among the highest-quality available for a CAD evaluation (e.g. scale, diagnostic algorithm, oversight for data quality, etc), the authors should provide more justification for using this modelling approach and the added value it provides for a CAD evaluation over focusing on the ~9,000 people with sputum test result data.

Thank you for this comment, and please additionally see the response to the Editorial comment above. Although the Reviewer is correct that TB prevalence surveys are designed to be “as accurate as possible”, they a necessarily limited by cost; indeed, the design of screening algorithm for prevalence surveys (symptoms or abnormal chest X-ray, followed by sputum microbiological investigation) means that the extremely large prevalence surveys needed to provide reasonable precision (usually +/-10%, as per the WHO Lime Book) are affordable. Sputum microbiological testing is the major driver of cost in prevalence surveys, and so programmes must necessarily limit the numbers of sputum tests done to make feasible; this is done by having a two-stage screening approach, recognising that some prevalent TB may be missed.

In our analysis we were able to utilise all data available for all the ~62,000 participants in the prevalence survey, rather than just the ~8000 who underwent a sputum microbiological test. In a programmatic prevalence survey or community-based active case finding intervention, CAD would be used as an initial screening test among all participants. As prevalence survey participants who underwent sputum testing have substantially different characteristics (see lines 254-265, and Table 1) and prevalence of disease compared to participants who did not undergo sputum testing, non-inclusion of these participants will bias estimates of accuracy. Therefore, we believe that the latent class modelling approach adopted here provides improved estimates of accuracy of CAD for the target population (community members participating in TB screening programmes), rather than focusing on only those whose sputum was tested. We have added additional text to LINE442-463 as per additional editor comment above to provide greater clarity to this point.

It is not clear from the manuscript text whether the CAD4TBv6 scores were as part of the Bayesian modelling. The authors should provide more details in the manuscript body about what variables were used to build the model to estimate TB prevalence.

The CAD4TBv6 scores are an independent predictor of participants’ latent TB status. Full details of the model structure and covariates used to estimate prevalence are provided in Lines 216-219, and in Supplemental Text 2.

*“Full model details and diagnostics are reported in Supplemental Text 2. We placed informative priors on the overall prevalence of TB, inferred by the prevalence survey results, and weakly informative priors on other model parameters, including presence of cough of greater than two weeks, age, sex, and history of previous TB treatment.”*

The authors should be more precise when writing about the approach. The analysis uses a modelled bacteriological reference standard. Sometimes the text reads as if the modelling does not exist. Example: Line 84 “…against the bacteriological reference standard used within the prevalence survey.”
Thank you. Throughout we have modified the text to ensure that we refer to TB model-estimated prevalence.

The procedures for reading CXR images are not totally clear. The text on Line 108 indicates “either one of the Clinical Officers”. Does each image get two independent reads from two different Study Clinical Officers? Or were there two Study Clinical Officers per day, with one read by either Officer per image?
Apologies that this was not clear.

Yes, each image received two independent reads from two different Study Clinical Officers. The CXR images were read by both clinical officers blinded to each other’s read. They both independently reported the findings in the three classifications of: A) normal; B) abnormal, suggestive of TB; or C) abnormal other. Line 140-143

*“Study Clinical Officers who had received intensive one-week training in CXR interpretation independently read each film, blinded to each other’s readings. In addition, one clinician was blinded to the participants’ symptoms. (Clinical Officer characteristic are summarised in Supplementary Text 1).”*

In the discussion about different software starting on Line 308, it should be noted that within each software new version are released on a regular basis. This analysis was done with a version of CAD4TB which is no longer commercially available. New users will receive CAD4TB v7.

Thank you for highlighting this. We have captured this on line 388-392 as follows:

*“Rapid advances have been made in CAD software development, with a total of 12 software solutions identified in March 2021 and version updates occurring frequently. (8, 28) For example, this analysis was done with a version of CAD4TBv6 software which is no longer commercially available. This has been replaced by a newer version CAD4TB v7 with reported enhanced analysis.(29)”*

Minor Comments:
Fig 1: The sum of groups under ‘Microbiological status assessed’ do not equal total (2,446 + 4,592 + 1,190 = 8,228). In the ‘Microbiologically-negative for TB’ box, fix the number formatting so it includes a comma.

Thank you for highlighting this. The summing of numbers is what is in the data (i.e. in the prevalence survey, it is possible some people got sputum testing who probably weren’t eligible. We have added a footnote (Line 253) to explain that more people received sputum testing than those with abnormal CXR/symptom abnormalities. We have also done formatting on figure 1 as noted.

I believe that references #6 and #16 are the same.

Thank you for highlighting this, we have corrected that.

**Reviewer #2:** This study addresses a very important topic, the accuracy of computer aided chest X ray screening of the general population, within a national prevalence survey in Kenya. The article is well written but it does need to address several major and minor points.

Thank you for these comments.

**Major points**

Being a diagnostic accuracy study, the article should adhere to the STARD statement and include the checklist as an annex indicating the pages where each item is addressed.

Thank you for this observation. We looked carefully at the STARD guidelines when analysing and reporting the study. However, as our study is not really a diagnostic accuracy evaluation but an analysis where we estimate test performance within a latent class modelling framework, it was not possible to clearly fit the reporting into this framework.

Although I agree the results are promising, the discussion should better address limitations and shortcomings. It seems to me to be a bit too optimistic and a healthy scientific skepticism would not reduce the value of findings and CAD for community based screening, but provide a more rigorous and objective interpretation of results. For example, in line 352, “highly accurate” without mentioning the low specificity in older age groups and previous TB history (% of those groups should be provided too). Same in line 369-361. Are you sure your results support all these benefits? How can false positive bacteriological tests be reduced without also considering false positive CXR results? Also, a more exhaustive discussion on the role of screening and testing persons with and without symptoms should be provided. Finally, comparing the results with the 2021 TB screening recommendations from WHO and saying if this study results support the recommendations or if authors suggest modifications.

Thank you for this input. We agree with your observation and we have enriched the discussion section with these reflections. Line

*Line 363-378 “The availability of portable/ultra-portable CXRs and CAD offer a potential solution to conduct community-based ACF for at risk groups in densely populated urban areas where TB transmission is now concentrated.(8) We have demonstrated that, overall within the prevalence survey population, CXR based screening in combination with CAD is accurate. However, due to low specificity in older age groups and persons with previous TB history, and adaptive CAD threshold could be more efficient than current approach of a single threshold for all participants. CAD gives the additional flexibility for TB programs to vary the threshold for sputum testing.(8) Given the limited resources available to National TB Programmes, by varying the CAD screening threshold, the number of TB cases deemed acceptable to be missed can be balanced against how much money is available to spend on expensive confirmatory sputum investigation.(8) By adopting an adaptive threshold within population groups, we believe that further gains in accuracy and programme efficiency can be gained. CXR and CAD as tools for community-based TB screening ACF, additionally offer the potential for individuals and TB programmes, including: earlier diagnosis; identification of asymptomatic TB, potentially reducing transmission; reductions in false positive bacteriological tests with harm from prolonged unnecessary treatment; and reduction in catastrophic costs.(8, 13, 28)”*

*Line 483-487 “Our findings support the 2021 WHO recommendations on use of CAD in place of human readers, in resource limited settings where radiologists are scarce, for interpreting digital chest X-rays for screening and triage for TB disease among individuals aged 15 years and older in populations in which TB screening is recommended.(5) However, we recommend an adaptive approach to setting screening thresholds as this could further improve screening accuracy and efficiency.”*

**Specific comments:**

Introduction

Line 65, I suggest to indicate the year of the WHO TB screening guidelines instead of “most recent”

Thank you, we have updated it to capture the year. Line 91.

Line 66, when authors say data come from “clinical settings” do they mean “and not from population based settings”? This could be specified.

Thank you. Yes, and we have now updated to be more specific. See Line 91-94.

*“However, supporting data have predominantly come from clinical settings and not community-based settings, and CAD diagnostic accuracy is likely to vary considerably across different screening strategies and populations. (6-8) ”*

Line 76, and specificity is probably lower than microbiological confirmation, is it not?

Thank you. We have included low specificity of CXR as one of the barriers-Line 100-103.

*“Barriers to widespread CXR use include low specificity, limited access to high quality radiography equipment, critical shortage of radiologists in low- and middle-income countries (LMICs), and inter-and intra-observer variations during interpretation.”*

Line 87, “..vary between population groups..” could authors specify which population groups. For example something like “… vary between general population and high risk groups or symptomatic groups..”

Thank you. We have now specified the population groups we were referring to as captured on line 115-119.

*“We hypothesized that CAD4TBv6 diagnostic sensitivity and specificity would meet the target product profile (TPP) for a test to identify people suspected of having TB, but that accuracy would vary between the general population compared to older age groups and people previously treated for TB , implying that an adaptive approach to CAD screening may add additional efficiencies to screening programmes.(19)”*

**Methods**

Line 93, authors could specify if the Kenya National TB prevalence survey followed WHO standard methods for prevalence survey and include the reference

Thank you for this important highlight. We have included this on line 124-125.
 *“The Kenya National TB prevalence survey followed WHO standard methods for prevalence surveys.(20)”*

The study population section does not describe the study population. Were they only adults? Representative at national level (or province level)? HIV positive and negative? After reading the full article we learn about these items, but the population should be described here

Thank you. We have updated the study population section to capture your inputs and clarify further.

Line 128-136

*“The Prevalence Survey (reported elsewhere) (17, 18) was a population-based national cross-sectional study conducted in 2015-2016.The target population for the survey comprised of all persons (male and female) aged 15 years and above, residing in Kenya and drawn from 100 clusters across the country. The aim of the survey was to determine the prevalence of bacteriologically confirmed pulmonary TB among adults aged 15 years and older and used the WHO-recommended screening strategy including symptom questionnaire and CXR.(20) HIV testing was not undertaken as part of the survey and participants self-reported their HIV status. There were 76,291 eligible participants out of whom, 63,050 (83%) were enrolled into the survey. Participation rates of females were higher than that of males at 87% and 77% respectively. (17, 18)”*

Line 105 What is meant by “independently”? Two readers? Not clear. Where readers also blinded to clinical information (symptoms/no symptoms, HIV status, TB contact, etc. But symptoms would be the most important). This comes back in the discussion line 420.

Apologies that this is not clear. Yes, each image received two independent reads from two different Study Clinical Officers. Each Clinician individually checked the quality of the radiographs and made an interpretation as either normal, abnormal suggestive of TB or abnormal other. The second Clinician assessed the symptom screening results and image interpretations to make an informed decision. Line 140-142.

*“Study Clinical Officers who had received intensive one-week training in CXR interpretation independently read each film, blinded to each other’s readings. In addition, one clinician was blinded to the participants’ symptoms.”*

Line 107 “..published definitions..” is insufficient for a study focused on X-ray reading. I suggest that you include a brief description of what was normal, abnormal. Etc

This is well noted. We have expounded on this on Line 146-157.

*Definitions*

*“In the Prevalence Survey protocol, Clinical Oﬀicer field interpretations of CXRs undertaken were defined as: a) “normal” if there were no identifiable cardiothoracic or musculoskeletal abnormality; b) “abnormal, suggestive of TB” if there was any infiltrate or consolidation, non-calcified nodules, cavitary lesion, pleural effusion, hilar or mediastinal lymphadenopathy, linear or interstitial disease; or c) “Abnormal other” if there were features of any musculoskeletal abnormality, cardiac abnormality, any other pulmonary and pleural abnormality not consistent with TB, diaphragmatic, costophrenic angle blunting not related to effusion, solitary calcified nodules or lymph nodes (18). In case of discordance between the two Clinical Oﬀicer readers on “abnormal, suggestive of TB,” versus “abnormal other” or versus “normal,” for the purpose of sputum eligibility the film was classified as “abnormal, suggestive of TB”; discordance of “normal” vs. “abnormal other” was classified as “abnormal other.”“*

Line 112 What if the person had no cough or could not produce sputum? Please clarify in the text

During the survey, participants were encouraged to produce sputum, for those with difficulties, walking and breathing exercises were performed. However, there were still some (approximately 6%) that did not produce sputum. It is not clear what happened in this case.

Line 161-164
*“A total of 9,715 (15%) participants were eligible for sputum collection. Participants were encouraged to produce sputum and were taken through some walking and breathing exercises. Two sputum samples (spot and following morning) were to be collected, out of the eligible participants, 9,120 (94%) had at least one sputum submitted.”*

Line 114 please revise spelling of Löwenstein-Jensen

Thank you, this has been revised.

Line 115 How were discordances between Xpert, culture and smear managed? Please report the frequency of discordancies and the algorithm or decisions taken when this occurred

Thank you for this question. Though smear, Xpert and culture tests were conducted on the sputum samples, the case definition for pulmonary TB was bacteriological confirmation by Xpert or culture. Therefore if a participant was Xpert, culture or both Xpert and culture positive, only then were they classified as bacteriologically confirmed. We have added a sentence on the case definition in line 177-179.

*“The case definition of a pulmonary TB case was one who was eligible for the survey and was bacteriologically confirmed - Xpert and/or culture positive for MTB. Though smear microscopy was conducted for all collected sputum, this was not included in the case definition.”*

Line 117-119 revise spelling of Mycobacterium (if it requires capitalizing, italizing, etc)
Noted and revised.

Line 121 and symptoms?
What is meant by Sputum Xpert and/or culture positive? Do you mean either sputum or Xpert or culture positive?. Same in lines 132-133

Apologies for this confusion. We mean either Xpert or culture positive, or both. Smear was not used as a case definition for pulmonary TB in the prevalence survey. Line 177-179.

Line 131 What is a “higher score”?
We have updated this to say scores closer to 100

Line 190-191

*Results were provided as probabilistic scores, ranging from 0 to 99, with higher scores closer to 100 indicating a greater probability of TB.*

Line 143 this is the first time that authors mention if sputum was collected or not. It should be mentioned earlier as it is a key aspect of study design.

This is noted and captured in the methods section Line 161-164

*“A total of 9,715 (15%) participants were eligible for sputum collection. Participants were encouraged to produce sputum and were taken through some walking and breathing exercises. Two sputum samples (spot and following morning) were to be collected; out of the eligible participants, 9,120 (94%) had at least one sputum submitted.*

*The sputum samples collected were transported under cold chain to the National Tuberculosis Reference Laboratory.”*

Line 146 please mention the primary study outcome earlier.
Thanks. We have updated the introduction section to capture the study outcomes earlier, see Line 110-115

*“Our primary study outcome was to use a Bayesian modelling approach to evaluate the accuracy (sensitivity, specificity, and area under receiver-operator curve [AUC]) of CAD4TBv6 and Clinical Oﬀicer CXR interpretation against a modelled bacteriological reference standard. Our secondary study outcome was to estimate sensitivity and specificity for participants by age group, sex, cough of more than two weeks status and history of previous TB treatment, summarised as a function of CAD4TBv6 threshold.”*

Why are predictive values not calculated, especially if the data comes from a population based study?

Thank you for this suggestion. The positive predictive value is highly dependent on the prevalence of TB in the target population, especially with a rare disease such as TB. Additionally, as TB prevalence varies considerably between population subgroups (particularly by age and sex), and some groups in the study are small, there would be considerable uncertainty around any PPV estimates.

In order to ensure results of our study are as generalisable as possible, and not over interpreted (i.e. readers may assume that PPV values reported in our study are transferable to other settings and populations), we have elected not to report these here, and instead report the test characteristics of sensitivity, specificity and AUC.

Line 148 The fact that cough more than two weeks was used as a criteria should be explained.

Thank you, the prevalence survey procedures and definitions were guided by the WHO Tuberculosis Prevalence Surveys Handbook. We have referenced this in our paper.

Line 159-161 *“Participants with a CXR classified as “abnormal, suggestive of TB” by either one of the Clinical Oﬀicers, or with a cough of more than two weeks or who declined CXR screening were eligible for sputum collection as per the survey protocol.”*

Lines 153-155 What is meant by “underlying status of active pulmonary TB” and by “underlying true TB status? Please clarify in the text

Thank you, this is the modelled latent class of TB status. As sputum examination was conditional on abnormal chest X-ray/presence of TB symptoms, it was necessary to estimate the underlying prevalence of TB among the study population through modelling.

Line 155 Model diagnostics should be reported in the results section

Thanks. Model diagnostics are reported on Line 304 and in detail in Supplemental Text 2.

*“Model Gelman-Rubin statistics were all <1.01 and trace plots showed good mixing across chains (Supplemental Text 2).”*

Line 158 this is the first time authors mention the combined reference standard. Earlier in the text, authors indicate sputum Xpert or culture positive.

Thank you. By combined we mean either Xpert and/or culture positive.

Line 160 what is cmdstanr interface.

Thank you. cmdstanr is an interface to run Stan models within R (<https://mc-stan.org/cmdstanr/>). We have added a reference to this in the manuscript. Line 223

*“Models were fitted in Stan using the cmdstanr interface,(22) with convergence assessed by inspecting trace plots across three sampling chains and Gelman-Rubin statistics.”*

Line 167 Which threshold?

Thank you. Here we refer to the CAD4TB threshold that would achieve an optimum sensitivity of at least 95%, as defined in the target product profile. Line 229-223

*“In secondary analysis, we restricted model sensitivity and specificity estimates for participants by age group, sex, cough of more than two weeks status and history of previous TB treatment, and summarised as a function of CAD4TBv6 threshold, estimating what accuracy would be achieved by setting an overall screening CAD4TBv6 threshold to achieve the optimum TPP sensitivity cut-off (95%).”*

Line 169 How can HIV status have missing data if testing was not performed? Please clarify.

Thank you for seeking this clarification. HIV testing was not done as part of the survey and the results captured under HIV were a) Self-reported b) For the bacteriologically confirmed as per case definition, once referred for TB treatment, they were offered HIV testing as part of standard of care.

*Line 133-134 HIV testing was not undertaken as part of the survey and participants self-reported their HIV status.*

*Line 182-184 As HIV testing was not undertaken as part of TB prevalence survey activities, in line with the national guidelines, participants referred for TB treatment were offered HIV testing at referral facilities.*

**Results**

Line 183 Field reader is not used before in the text.

Apologies, now replaced with “Clinical Officer”

Figure 1 the flowchart provides new information that was not clear in the methods. Why is only a fraction of those assessed by CXR evaluated with microbiology?

Apologies that this was not clear. Only those eligible for sputum as per the prevalence survey procedures i.e. abnormal CXR suggestive of TB and/or have cough more than two weeks were eligible for sputum testing.

Line 159-161 in the Kenya TB prevalence survey procedures, we have captured this “ *Participants with a CXR classified as “abnormal, suggestive of TB” by either one of the Clinical Oﬀicers, or with a cough of more than two weeks or who declined CXR screening were eligible for sputum collection.”*

Why is smear not reported, while it is mentioned at least twice in methods?
Thank you. Even though smear was done as part of the prevalence survey, due to the low sensitivity, it was not included in the case definition for pulmonary TB, as previously mentioned was Xpert and/or culture positive only were used.

Line 177-179.

*The case definition of a pulmonary TB case was one who was eligible for the survey and was bacteriologically confirmed - Xpert and/or culture positive for MTB. Though smear microscopy was conducted for all collected sputum, this was not included in the case definition.*

Please report data for narrative results presented in lines 197-199.
This has now been updated see line 260-262.

“Compared to participants with CXRs classified by Clinical Officers as “normal” or “abnormal, other,” participants with CXRs classified as “suggestive of TB” were more likely to be men (50.5%), and have been previously treated for TB (50.3%).”

Line 202 The p<0.0001 is for each comparison?

Thank. No, this p-value is for the omnibus test across all three categories.

Table 1. What about missing data? Is the p for age groups a p for trend? Please specify.

Variables with missing data are reported in the Table (only self-reported HIV status had missing values). P-values are for omnibus tests across all three groups.

Line 280 first time chronic cough is mentioned. If you mean cough >2 weeks, please say so for consistency within the text.

This is well noted and we have corrected the text to reflect this.

Table 2, the title is not self-explanatory and the subtitles are not clear. Credible intervals were not mentioned in the methods.
This is noted. We have added the credible intervals in the statistical analysis section and worked on the table 2 titles. (Line 320-322)

Line 204-205 *“We summarised posterior distributions of model parameters using posterior means and 95% credible intervals (CrI).”*

**Discussion**
Line 332. Xpert is highly specific in asymptomatic patients with low pre- test probability?

Thanks, yes, we have referenced the paper by Marks et al (NEJM 2021)

Line 337 and 339, please indicate the sensitivity and specificity (in a rounded %).

This is well noted, the sensitivity and specificity ranges have been included.

Line 351-354*: Very high sensitivity of 98-100% was demonstrated in participants in older age groups (41 years or older), those with reported cough>2 weeks and participants with previous TB history. Conversely, participants in older age groups and those with previous TB history had lower specificity ranging from approximately 38-70%.*

Line 353, it is not clear how 50% of Xpert tests would be saved.
Thank you for highlighting this. This was in reference to a paper by Qin et al, we have removed this for this context.

Line 363-364, this is not mentioned in methods (representative of the general pop and random selection). Same comment for line 397.

Thanks for this. We have updated the methodology section to give more details of the prevalence survey methodology.

Line 128-136 *“The Prevalence Survey (reported elsewhere) (17, 18) was a population-based national cross-sectional study conducted in 2015-2016.The target population for the survey comprised of all persons (male and female) aged 15 years and above, residing in Kenya and drawn from 100 clusters across the country. The aim of the survey was to determine the prevalence of bacteriologically confirmed pulmonary TB among adults aged 15 years and older and used the WHO-recommended screening strategy including symptom questionnaire and CXR.(19) HIV testing was not undertaken as part of the survey and participants self-reported their HIV status. There were 76,291 eligible participants out of whom, 63,050 (83%) were enrolled into the survey. Participation rates of females were higher than that of males at 87% and 77% respectively. (17, 18)”*

Line 401, what was anticipated?
The design of prevalence surveys is to ensure high sensitivity by the CXR readers. We therefore anticipated a high sensitivity and low specificity, however our findings showed a lower sensitivity and high specificity.

Line 418-424  *“In prevalence surveys, image classification criteria are set to a low threshold for referral for sputum testing, and non-expert readers like Clinical Oﬀicers are trained to interpret with higher sensitivity and lower specificity to avoid missing prevalent TB cases.(20) We found that, overall, the sensitivity of Clinical Officer CXR interpretation (“suggestive of TB”) for the underlying true TB status was lower (44%) and specificity was higher (89%) than anticipated,(5) but that sensitivity was substantially higher (84%) and specificity lower (54%) among participants with previous TB, and with no appreciable differences by other participant characteristics.”*

Line 426. The fact that limitations are that results are dependent on the model assumptions is a generic statement. Which model assumptions do authors have doubts upon?
Thank you for pointing this out. We have removed this from the limitations.

Line 430-431. This is not consistent with what is described in the methods. Extra pulmonary TB has not been mentioned at all earlier in the text.
Thank you for highlighting this. We have now removed this from the discussion.

Please include a discussion on why women were overrepresented in the survey.

Thanks*.*

Line 382-384 *“ The higher participation rate in women is a well-recognised finding across prevalence surveys that have been conducted in recent years.(29) This is possibly due to men being less likely to be at home when prevalence survey activities are undertaken.”*

In general, the impact of limitations on the study results should be further discussed.

Thank you. We have expanded upon the limitations. Line 465-478

*“Limitations include our study participants being aged 15-years and above, therefore we cannot comment on the diagnostic accuracy of CAD4TBv6 in children. Additionally, the study only included bacteriologically-confirmed TB; we did not undertake the assessment of accuracy in participants with sputum negative TB (clinically diagnosed). We also were not able to stratify performance by HIV status as testing was not systematically conducted during the prevalence survey. Self-reporting of HIV status in the prevalence survey may not be accurate and is a limitation of the parent survey from which we obtained data for this analysis. In high TBHIV burden countries like Kenya, it will be important to consider HIV testing as part of the TB prevalence surveys in future. This would have been important for an in-depth subgroup analysis in a high TB-HIV prevalence setting. Kenya has a HIV prevalence of 4.9% with approximately 1.6 million people living with HIV and an estimated HIV-positive TB incidence at 70/100,000.(1, 36) We therefore expected a lower CAD specificity in our setting as CXR is known to be less sensitive in immunocompromised patients with pulmonary TB.(37-40) We recommend further evaluation of CAD software in high TB-HIV prevalence settings and further studies on accuracy within HIV-positive populations.”*

The fact that HIV was not tested is a major limitation. Results could also be stratified by HIV positive by self-report, as well as by symptom status among them (and considering 14 days of cough is used, instead of only 1 day).

Thank you for your suggestions. We agree that self-reporting of HIV status in the prevalence survey may not be accurate and is a limitation of the parent survey from which we obtained data for this analysis. In high TBHIV burden countries like Kenya, it will be important to consider HIV testing as part of the TB prevalence surveys in future. Apologies, we are not clear on the stratification suggested.
