# Supplementary material for: Accuracy of computer-aided chest X-ray in community-based tuberculosis screening: Lessons from the 2016 Kenya National Tuberculosis Prevalence Survey
Source: PLOS Glob Public Health. 2022 Nov 23;2(11):e0001272. doi: 10.1371/journal.pgph.0001272 (PMC10022380; doi:10.1371/journal.pgph.0001272)
Supplement: Attachment — Submitted filename: response to reviewers 14th july 2022_pm.docx [file pgph.0001272.s004.docx]

**PGPH-D-22-00030
Accuracy of computer-aided chest X-ray screening in the Kenya National Tuberculosis Prevalence Survey
PLOS Global Public Health**

Thank you to members of the PLOS Global Public Health Editorial Board for highlighting that our manuscript may be of interest to the readers and for the opportunity to address points raised by Reviewers. Please find below a point by point response to reviewers. Please also find attached the manuscript with track changes and the clean version. The line numbers on the responses are from the un-tracked version of the manuscript. We look forward to your feedback.

**Editor comment:**

Please review your reference list to ensure that it is complete and correct. If you have cited papers that have been retracted, please include the rationale for doing so in the manuscript text, or remove these references and replace them with relevant current references. Any changes to the reference list should be mentioned in the rebuttal letter that accompanies your revised manuscript. If you need to cite a retracted article, indicate the article’s retracted status in the References list and also include a citation and full reference for the retraction notice.

We have reviewed the references and made a few edits on the WHO naming. No references have been retracted nor have we added any reference.

**Response to reviewers July 2022**

**Reviewer #3:** The current title does not seem to add to the body of knowledge as you have already stated in the manuscript about the 2021 WHO recommendations on the use of CAD in place of human readers, in resource limited settings where radiologists are scarce, for interpreting digital chest X rays for screening and triage for TB disease among individuals aged 15 years and older in populations in which TB screening is recommended. However, the recommendation of “an adaptive approach to setting screening thresholds as it could further improve screening accuracy and efficiency” seems to be the key finding.
It would more beneficial if the title could be tweaked. The manuscript seems to be talking to something like this “Optimizing community-based TB screening accuracy and efficiency in high burden countries ( or resource limited settings): Lessons learned from the Kenya National TB Prevalence survey”

Thank you for this suggestion. We have revised the title to- **Accuracy of computer-aided chest X-ray in community-based tuberculosis screening: Lessons from the 2016 Kenya National Tuberculosis Prevalence Survey**

The methodology and result sections are not clear. They appear ambiguous and not well presented. There are a lot of findings but not well organized to have a good thought flow and there are a lot of repetition of same information in different ways.

Thank you for this comment. We have reviewed the Methods section in detail, and are confident that the methods are reported clearly and concisely. Should the Editor have specific comments that they require us to address, we would be very happy to do so.

**Reviewer #4:** The paper explores the use of CAD and CXRs on screening for active TB using secondary data from a large national TB survey.

The objectives of the study, and the methods including the statistical methods are clearly described and appropriate. Listed below are are few comments, that can be discussed further by the authors.

Thank you for the feedback on the objectives and methodology.

1. The aim of screening programmes is to pick up any suspected cases and do further investigations. In this case sensitivity becomes much more important than specificity because missing active cases of TB is more consequential and grave than misclassifying, negative individuals. The authors compared optimum and minimum TPP, within this range is there are 'desirable' cut off TPP that is clinically, economically etc preferable?

Thank you for this question. For this study, we used the WHO Target Product Profiles for a community screening test which are consensus standards for accuracy and implementation (*High-priority target product profiles for new tuberculosis diagnostics: report of a consensus meeting. Geneva, Switzerland: World Health Organisation; 2014. Contract No.: WHO/HTM/TB/2014.18.)* The desirable threshold as captured in the discussion section line 375-384 is a trade-off between the numbers of TB cases deemed acceptable to be missed balanced against how much money is available to spend on a highly sensitive confirmatory sputum investigation (e.g. Xpert). During country roll out of CAD, STOP TB partnership has offered different ways to get a “desirable working threshold” including iterative threshold score calibration. We addressed these issues in the discussion.

**Line 375-380**

*CAD gives the additional flexibility for TB programs to vary the threshold for sputum testing.(8) Given the limited resources available to National TB Programmes, by varying the CAD screening threshold, the number of TB cases deemed acceptable to be missed can be balanced against how much money is available to spend on expensive confirmatory sputum investigation.(8) By adopting an adaptive threshold within population groups, we believe that further gains in accuracy and programme efficiency can be gained.*

2. The authors did mention other important considerations before considering implementing the CAD system. The proposal or goal will be to see this being used middle to low income countries as well. The authors should consider in the discussion, what the main challenges of implementing this might be. It might be easy to get CXRs using portable machines, but is it easy to transmit/upload the images to a central place/server where these can the be read? Are there connectivity issues etc.

Thanks for this. The advantage with most CAD software, (including the CAD4TB we used), there is an option of offline reading of the images using the CAD boxes. We have now added text to include this in the Discussion.

**Line 397-402**

*As national TB programs adopt CAD technology into screening activities, in addition to performance, other implementation considerations include; cost effectiveness, compatibility of the X-ray systems, input image format, connectivity requirement and offline operation option, integration with any patient archiving systems, customer service and support, data protection, and ability to detect other non TB conditions.(8, 30, 32)*
